# Predicting financial losses due to apartment construction accidents utilizing deep learning techniques

**DOI:** 10.1038/s41598-022-09453-w

**Published:** 2022-03-30

**Authors:** Ji-Myong Kim, Junseo Bae, Hyunsoung Park, Sang-Guk Yum

**Affiliations:** 1 Department of Architectural Engineering, Mokpo National University, Mokpo, 58554 South Korea; 2 School of Computing, Engineering and Physical Sciences, University of the West of Scotland, Paisley, PA1 2BE UK; 3 Department of Mechanical and Civil Engineering, University of Evansville, Evansville, IN 47722 USA; 4 Department of Civil Engineering, Gangneung-Wonju National University, Gangneung, 25457 South Korea

**Keywords:** Engineering, Civil engineering

## Abstract

This study aims to generate a deep learning algorithm-based model for quantitative prediction of financial losses due to accidents occurring at apartment construction sites. Recently, the construction of apartment buildings is rapidly increasing to solve housing shortage caused by increasing urban density. However, high-rise and large-scale construction projects are increasing the frequency and severity of accidents occurring inside and outside of construction sites, leading to increases of financial losses. In particular, the increase in severe weather and the surge in abnormal weather events due to climate change are aggravating the risk of financial losses associated with accidents occurring at construction sites. Therefore, for sustainable and efficient management of construction projects, a loss prediction model that prevents and reduces the risk of financial loss is essential. This study collected and analyzed insurance claim payout data from a main insurance company in South Korea regarding accidents occurring inside and outside of construction sites. Deep learning algorithms were applied to develop predictive models reflecting scientific and recent technologies. Results and framework of this study provide critical guidance on financial loss management necessary for sustainable and efficacious construction project management. They can be used as a reference for various other construction project management studies.

## Introduction

To solve the housing shortage in the city center, the construction of apartment buildings, is rapidly growing. High-rise and large-scale apartment construction are increasing the difficulty of construction work. As a result, due to various accident risk indicators that occur inside and outside of a construction project, the risk of financial loss due to accidents occurring in the construction site is quickly snowballing. Uncertainty about the loss prediction is also swelling^1^. For an efficient financial management of construction projects, the demand for a more methodical and reliable loss prediction model is increasing. However, existing risk assessment methods for construction projects do not keep pace with the needs as they rely on qualitative assessment based on experience or knowledge of the construction manager and views of clients and individual contractors^2^. Moreover, such quantitative evaluation method is challenging to reflect rapidly changing risks of construction work to be evaluated^2,3^.

Specifically, construction work has a higher risk of safety accidents because there are more outdoor works than indoor works. The difficulty of work is also greatly affected by environmental and geographical indicators. For this reason, among various industries, construction industry is classified as an industry with the highest risk of accidents^4^. For instance, the safety accident rate at construction sites is cumulative own to the use of heavy equipment and work at heights due to increasing size and height of buildings. In addition, there is a high risk of third-party damage to nearby buildings and pedestrians due to falling objects at building construction sites in the city center^5^. Noise and vibrations generated from construction sites are also causing problems. Furthermore, recent construction works in mountainous and river areas have experienced a lot of accidents due to geographical disadvantages. These factors are causing enormous economic losses despite the rapid development of new technologies and construction technologies of the fourth revolution such as Internet of Things, unmanned transportation, robot engineering, and 3D printing^6^. In addition, the severity and frequency of losses caused by natural disasters such as increases of severe weather and extreme weather events due to climate change are expected to gradually increase^7^. Therefore, it can be seen that the construction industry, which is highly affected by environmental and geographical factors, is exposed to the increasing risk of natural disasters. To reduce the vulnerability of these construction sites, the government is promoting active policies and systems. The private sector is also making efforts to make corresponding investments. However, the construction industry is still having the highest accident rate^1,6,8^. Thus, it is necessary to predict possible accidents and losses in the construction site in advance to establish a strategy for reducing the risk of financial loss and transferring the risk^9–13^.

Hence, the aim of this study is to generate a model that can systematically predict monetary loss of apartment construction sites due to accidents based on empirical data. In other words, this study intends to develop a model for predicting the financial loss of apartment construction sites associated with accidents using real loss data and deep learning algorithms at apartment construction sites. These frameworks and models can be applied to various construction projects in the future. They are anticipated to advance risk management of construction projects.

## Literature review

### Risk analysis of construction

Risk analysis of construction projects, can establish strategies to efficiently use limited resources in construction projects and ultimately, lead to successful construction projects by preventing risks and reducing damages in advance, to manage potential risks at construction sites^14^. However, risk analysis is less active than other construction management elements (i.e., schedule management, quality management, cost management, contract management, safety management, etc.)^1,2^. Moreover, regarding research methodology, quantitative methodologies are suitable in terms of efficient distribution and use of limited resources, which are the ultimate purpose of risk analysis of construction projects. However, qualitative methodologies are mainly adopted for risk analysis of construction projects customarily. Quantitative methodology can be used to develop a quantified model by identifying potential risk factors of construction sites by analyzing objective data and defining the weight of identified factors^15^. Despite these advantages, the reason that qualitative methodologies are widely adopted in risk analysis for construction projects is that it is difficult to introduce quantitative methodologies due to the complexity and specificity of a construction work^1,2^. This is because many construction contractors for each process have the same simultaneity to complete work at a fixed place within the contract period. Since there are many external works, there is a large amount of uncertainty own to the influence of various factors^16^. Therefore, when considering the uncertainty and specificity of a construction work, quantitative methodologies are more appropriate than qualitative methodologies for risk prediction. In addition, in order to reduce the uncertainty of a construction project and reflect the specificity of the construction project, risk assessment and prediction through a quantitative analysis method based on a scientific approach and statistical analysis of data are compulsory^17^.

Nevertheless, qualitative research methods are generally employed in risk analysis and evaluation of construction projects^18^. For instance, Baker et al.^19^ have pointed out that experiences and subjective opinions of clients, engineers, and related experts are mainly used in the construction risk assessment technique. In addition, in construction risk analysis and evaluation studies, risks are identified and evaluated by subjective evaluation indicators in addition to experience and expertise^3^. Moreover, risk assessments are sometimes made based on the experience and knowledge of experts through checklists or surveys of experts^20^. The reason why qualitative research methods are often used in risk analysis and evaluation of construction projects is because sensitivity analysis is mainly borrowed or risk measures are often used due to the lack of reliable data and limitations in data collection in risk analysis of construction projects^21–25^. Therefore, to increase the reliability and accuracy of risk analysis and evaluation of a construction project, it is important to collect and aggregate data that reflect actual losses. As shown in previous studies, various methods have been proposed for risk assessment and analysis of construction projects. However, qualitative research techniques based on the experience and knowledge of experts were largely used. Consequently, in order to analyze and evaluate risks of construction sites scientifically and objectively, quantitative evaluation based on numerical and reliable data is required.

### Assessing natural disasters for construction works

Due to recent increases in the severity and frequency of natural disasters, the loss is swelling. Moreover, the loss caused by natural disasters is expected to surge further due to increases of abnormal climate and severe weather caused by global warming^7^. To prepare for such losses, many countries, private organizations, and companies have developed tools for assessing the risk of natural disasters. For instance, HAZUS-MH for multi-hazard risk assessment developed by the US Federal Emergency Management Agency (FEMA) is a representative natural disaster risk assessment model that evaluates losses caused by natural disasters such as earthquakes, floods, and hurricanes^26^. Florida Public Hurricane Loss Prediction Model (FPHLM) developed by the state of Florida in the United States is a symbolic natural disaster risk assessment model for predicting financial loss and human damage caused by hurricanes^27^. Probabilistic Risk Assessment in South America and the New Multi-Hazards and Multi-Risk Assessment Method (MATRIX) in Europe are developed to predict economic losses due to natural disasters and to prepare for and reduce losses. These models are being used to evaluate potential risks (e.g., casualties and direct and indirect damage to buildings and infrastructure) from natural disasters such as hurricanes, floods, and earthquakes. Moreover, these models can utilize Geographic Information System (GIS) to integrate various geospatial and social data to evaluate more diverse and comprehensive potential risks^28^. Although these models can calculate the damage caused by natural disasters for buildings and ancillary facilities, social infrastructure, and human damage, they do not cover damage to construction works.

As a loss evaluation model for construction works, models of insurance companies and reinsurance companies are representative ones. For example, Swiss Reinsurance Company's Project Underwriting Management Application (PUMA), Munich Reinsurance Company's Munich Re Engineering Expert Tool (MRET), and Willis Re's engineer are demonstrative models. Through these models, relevant companies can calculate risks of insurers and potential customers and use them in consulting to reduce potential risks of customers. These loss assessment models can help insurance companies engineering underwriters and customers understand and calculate potential risks of construction. On the other hand, since these models were designed to be used in the insurance business taking existing customs of the insurance market into account, it is difficult to use them to calculate risks of pure construction projects^29^.

In addition, risk modeling specialists (e.g., Risk Management Solution, EQECAT, Applied Insurance Research, etc.) offer commercially available models for risk assessment of construction projects^28,30^. Since these models are commercial models, they are not easily accessible to the general public because their use is limited. The use of them is accompanied by a usage fee. Moreover, these models were developed for some developed countries. Therefore, there are many countries that these models do not cover.

### Evaluation according to construction type

A number of studies have been conducted for each type of construction work to predict the damage to construction works due to natural disasters. Some researchers have conducted studies to identify possible risks (e.g., weather conditions, equipment risks, material shortages, traffic conditions, health and safety, contract problems, labor shortages, etc.) in the infrastructure^31–34^. Yum et al.^15^ have used several natural disaster features such as rainfall and wind speed to identify indicators affecting the amount of damage and presented a methodology for estimating the amount of damage caused by tunnel construction. In addition, studies have been conducted to determine the frequency and severity of possible risks of building construction and to calculate the risk of loss using indicators of various categories such as natural disasters, geographic information, and construction information^6,35^. Kim et al.^14^ have proposed a model for computing plant construction loss risk. In their study, indicators affecting the risk of loss were recognized for indicators of numerous groups and a model for calculating the risk of plant construction loss through multiple regression analysis was offered. In one study, a number of risk indicators were defined and a calculation methodology was presented through multiple regression analysis to develop a model to predict repair costs of an international hotel chain caused by natural disasters such as hurricanes, floods, lightning strikes, and rainfall^29^. As mentioned above, a number of studies have been conducted to calculate the loss of construction work due to natural disasters by the type of construction work. Since the construction environment, geographical conditions, safety, and worker training level are very different for each type of construction work, major risk indicators differ for each type of construction work^36^. Therefore, in order to predict the loss of apartment construction due to accidents, it is essential to identify risk indicators for apartment construction based on reliable data and develop a predictive model.

### Analysis for improving safety and preventing the risk of accidents for construction project

Accidents during construction are fatal. Despite significant improvements in safety management over the past decades, accidents during construction still occur frequently, and a large amount of money is spent to deal with the accidents^37^. The many factors caused accidents and the relationships between them make safety management challenging^38^. Therefore, many existing studies have introduced new frameworks and technologies to analyze and evaluate risk identification and develop effective safety procedures. First, Betsis et al.^39^ analyzed accidents to identify the associations between various accident characteristics. The methodological approach involves collecting incident-related data, performing descriptive analysis, and corresponding cataloging of accessible data. This study creates a database to house all relevant data and identifies potential trends within the incident sample. The results highpoint the most common accidents associated with accidents related to construction work while also identifying correlations between the various parameters associated with them. Thus, the findings can help reduce the number and severity of work-related accidents. Management's commitment to safety and improved construction site organization with frequent inspections can reduce accidents. Second, Allison et al.^37^ provide a methodology to calculate the costs of accidents. A 'post-event' approach has been used to determine the average cost of an incident. However, these costs are highly volatile and may differ significantly from the baseline estimate depending on the nature of the incident. Thus, this study used clear scale identification to facilitate internalization of accidents' actual cost and positively inform safety program improvement. Third, Cabello et al.^38^ used the association rule method of data mining to extract knowledge from historical data of construction accidents. This study analyzed accidents notified via electronic systems, segmenting the data to investigate accidents at each stage of construction. The results reveal patterns and factors with multiple relationships at all stages of the construction. The findings support a framework for improving safety practices, providing a valuable reference for practitioner involved in the construction to improve risk management, preventive measures, and action plans. The following is an example using the Internet of Things (IoT). The IoT is used to improve data collection and information management at a construction site. Emerging technologies are being incorporated at all stages of the project lifecycle, especially during construction. Therefore, integrating technology and safety knowledge is crucial, including real-time decision-making capabilities and knowledge management about risk. In this study, Martinez-Rojas et al.^5^ built an IoT infrastructure integrated with open source libraries to support expert decision-making. Finally, Lestari et al.^40^ developed a framework to evaluate the safe environment and improve construction. This study collected and analyzed quantitative and qualitative data. The results reflect, among other things, several issues related to perceived conflicts between construction and safety logic, cost trade-offs over other competing project priorities, unsafe communication, poor working conditions, and accepting poor safety as the norm. Based on these results, a new integrated safety environment framework was proposed to improve the safety performance of the construction industry. Despite many efforts to improve safety, such as the studies above, construction remains one of the riskiest industries. Therefore, continuous efforts and research on construction safety must be made.

## Research aims and methods

The purpose of this study was to develop a model to predict the monetary loss of an apartment construction site utilizing a deep learning algorithm based on fiscal loss data due to accidents at apartment construction sites. Detailed objectives of this study were: (1) to collect data on financial loss that occurred at real apartment construction sites; (2) to develop a loss prediction model through a deep learning algorithm based on collected data; and (3) to validate the deep learning algorithm model by comparing prediction results with other model results. For model validation, results of the deep learning algorithm model were compared with results of a multiple regression analysis model universally adopted for prediction.

**Figure 1:**
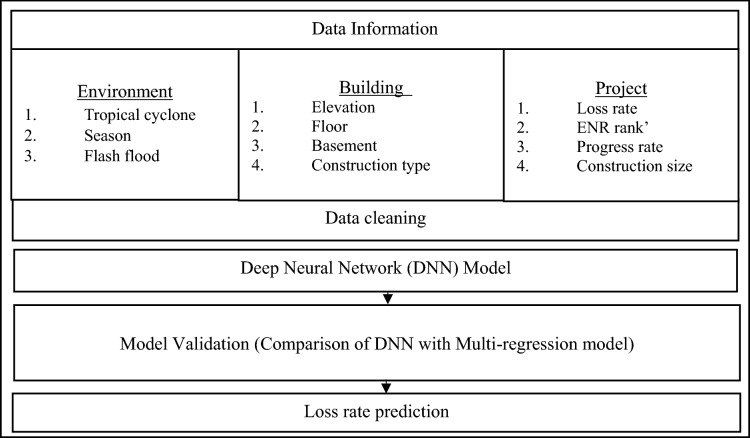
Workflow of the model.

The study was conducted in the following order as seen in Fig. 1 below. First, input and output variables associated with economic loss of apartment construction sites were gathered. Second, a deep learning algorithm model and a multiple regression analysis model were developed using input and output variables separately. Finally, the mean absolute error (MAE) and square mean square error (RMSE) values of these two models were calculated individually. Results of the two models were then compared. The deep learning model was developed using Python 3.7 and the multi-regression analysis model was established using IBM SPSS (Statistical Package for the Social Sciences) version 23.

## Data collection

This study adopted claims payout records of Contractors' all-risks insurance (CAR) from major Korean insurance companies to reflect financial losses of apartment construction sites. As for claims payout records, the amount was calculated through an objective analysis by a certified loss adjuster. The payment procedure was standardized and the reliability of the data was very high^6^. By using claims payout records, the actual financial loss occurring at the construction site of apartment buildings was objectively reflected and reliable results were derived. CAR is an insurance that indemnifies the contractor for economic losses such as structure, third party, equipment, material, and human damage that may occur at all phases of a construction project. CAR is applied to a wide range of construction works such as buildings, plants, roads, ports, bridges, and tunnels. It covers the entire period of construction from the start of the work to the commissioning stage^35^.

### Data description

Collected data for modelling were from different sources. Financial loss and related information were claim payout records extracted from different insurance companies’ databases. Allison et al. considered the indirect cost and indirect cost of the accident^37^. Due to the unavailability of non-insured loss related data, such as fines related to violation of safety measures at construction sites and another indirect cost of the accidents, in this paper insurance claims, pure cost of claims, which excludes conditions associated with an insurance policy from each company, was only considered, to avoid difference discrepancy in the data from different insurance companies.

The total number of losses was 930. None of the collected information contained personal information. The same dataset contains the construction details: the total construction cost of the project, construction period, construction site location, date of the accident, amount of loss, and detailed loss history. The number of floors of a building has a contribution in increasing the risk of falling objects; as the height increases the use of scaffolding and cranes is unavoidable which induces the risk of falling objects^5^.

In addition, claim payout data set contains natural hazard related variables such as, elevation, risk rank of tropical cyclone, risk rank of flash flood, and season. The specific information of the apartment construction site (i.e., location, loss classification, rank of Engineering News Record (ENR), number of floors, number of basements, total construction period, construction type, construction size, progress rate) were also collected. The natural disaster risk information was considered based on the location information of the collected data. In order to objectively and scientifically reflect natural disaster risk, the Natural Risk Assessment Network (NATHAN) of Munich Reinsurance Company was utilized to define several natural disaster risk levels. NATHAN is an online natural disaster risk level system that displays levels of risks of various natural disasters around the world, such as earthquakes, floods, lightning strikes, tsunamis, and typhoons. The natural disaster risk level of this system is independent and dependable as it is a synthesis of various studies and data such as simulation tools, the frequency and severity of past natural disasters, and public data^15^. The risk rank of tropical cyclone and risk rank of flash flood were identified using the location information of the construction site in this system. Elevation, ENR rank, floor, basement, total construction period, and progress rate were entered as numeric variables. Risk rank of tropical cyclone, risk rank of flash flood, season, location, loss classification, construction type, and construction size were also considered as nominal variables. A detailed description of each indicator is given in Table 1. As seen in Table 2, the dependent variable was the loss ratio divided by the total cost of construction and transformed to natural logarithm for normal distribution of data. The dependent variable was the loss ratio divided by the total cost of construction. The loss rate was transformed to a natural logarithm to make the data normally distributed. Variables were grouped into three groups: financial information which is the output variable, environmental-related information, and building and location-related information’s, which are input variables. The last ones have a direct effect on construction projects^41^.

**Table 1:** Variable description.

	Variable	Description	Unit
Financial information	Loss rate	The loss rate is the amount of loss incurred in the construction project divided by the total construction cost of the construction project	Real number
Natural hazard-related information	Elevation	Elevation of the loss-occurring construction site (m)	Real number
	Tropical cyclone	The tropical cyclone risk class of the construction site is classified into Zone 0–5 (based on the maximum wind speed of the 100-year return period)	Nominal (0–5)
	Flash flood	The flash flood risk class of the construction site is categorized into Zone 1–6	Nominal (1–6)
	Season	Seasonal classification at the time of loss	Nominal 0: fall 1: Spring 2: Summer 3: Winter
Construction site and apartment information	Location	Classification according to the location of the construction site where the loss occurred	Nominal 1: Suburban 2: Urban 3: Metropolitan
	Loss classification	Classification according to the occurrence of loss	Nominal 0: Object 1: Third-party
	ENR rank	The rank of Engineering News-Record (ENR)	Numeral
	Floor	Number of floors	Natural number
	Basement	Number of basement floors	Natural number
	Construction type	Classification by type of construction work	Nominal 1: reinforced concrete work 2: steel framework 3: other work
		Elevation of the loss-occurring construction site (m)	Real number
	Construction size	Classification according to the total construction cost	Nominal 1: Small-scale sites with less than 2 billion KRW 2: Medium-sized sites with 2–12 billion KRW 3: Large-scale sites with more than 12 billion KRW
	Progress rate	Process rate at the time of loss (%)	Real number
	Total construction period	The total period of construction work (month)	Real number

**Table 2:** Descriptive statistics.

Variables	N	Mean	Minimum	Maximum	Std. deviation
Ratio	930	2.43	− 2.70	7.43	1.78
Elevation	930	45.52	0.00	792.00	50.87
ENR rank	930	42.12	1.00	100.00	43.01
Total construction period(month)	930	25.35	5.00	126.00	16.30

The variable season changes and affect change in other natural hazards related variables such as flood tropical cyclone which occurs during a given season. As the seasons differ their effect on accidents also differ; summer and spring were associated with higher number of accidents^42^. Ahmed established a very long list of causes accidents at a construction site in details^43^. Lee, G et al. identified that the working elevation, weather, construction size etc. contribute in accidents^44^. The personal information of the claimant such as age, year of experience, and type of accident was not used though there is a likelihood of an increase in the cost of accident associated with this information^39^.

The cost of accidents such as penalty and legal data were difficult to find and were not taken into account^41^. Safety measures of the construction site and injured body part of the claimant, whether safety-related protection such as helmet and other related safety protection data were not available in the dataset, so they were not used in this study^40^.

## Implementation of deep learning algorithm model

Nanotechnology, robotics, 3D printing, big data, unmanned transportation, artificial intelligence, and so on referred to as the 4th revolution technologies are recognized as new paradigms. Their use is rapidly increasing as they are converged in innumerable industrial fields^45,46^. In order to reduce and prevent the risk of financial loss due to the specificity and uncertainty of construction projects and to break away from the stigma of being a traditional risky industry, active introduction of a new paradigm is essential. Moreover, several technologies such as ICT (Information and Communications), sensors, and IoT (Internet of Things) recently announced for construction sites not only can promote user safety, efficiency, and convenience, but also can accumulate huge amounts of data. Since the use of deep learning technology is inevitable to analyze such vast amounts of big data, the demand for deep learning technology is expected to further surge. Therefore, this study proposes a framework for developing deep learning technology for the analysis of big data related to construction projects. It tries to contribute to the reduction and prevention of financial losses that may occur in future construction projects by developing predictive models.

Deep learning technology is a kind of machine learning technology for regression and type classification of input data. A model is formed using various components such as hidden layer, output layer, input layer, neuron, activation function, and weight. Since a combination of these various components is possible, deep learning technology can be used for various data. Own to these various arrangements and applicability, it is used in countless industries and academia. It is predominantly and widely accepted in recognition and prediction fields^45,46^. Deep learning algorithms are typically classified into Convolutional Neural Network (CNN), Auto Encoder (AE), Generative Adversarial Network (GAN), Recurrent Neural Network (RNN), and Deep Neural Network (DNN) according to their processing scheme and configuration. For instance, DNN is a symbolic neural network with voluminous combinations and number of hidden layers. It is mostly approved to model and train complex nonlinear interactions^47,48^. DNN can implement artificial neural networks of various structures. It is broadly adopted for prediction and classification in numerous areas^49^. Considering the versatility of the model and features of input and output data, this study employed the DNN algorithm and proposed a model framework for predicting economic losses of apartment construction projects.

The established model was confirmed using Root Mean Square Error (RMSE) and Mean Absolute Error (MAE) as representative evaluation indicators of artificial neural network models^50^. Since both RMSE and MAE values calculate the deviation between actual and predicted results, they are suitable for expressing the precision of the model. RMSE is a method of expressing the residual between the actual value and the predicted value of a model as one measure. The greater the RMSE value, the greater the prediction error. MAE is an absolute value that is averaged by adding residuals of the actual value and the predicted value of a model. The higher the MAE value, the higher the prediction error. Of total data, 70% were nominated as learning data and the remaining 30% were adopted as test data. Of the learning data, 30% were designated as validation data. The input data was preprocessed utilizing the z-score normalization method to normalize data distribution.

### Setting of DNN model

In order to make an optimal model of the DNN algorithm, it is important to discover the optimal network structure scenario and hyper-parameter tuning for the data over trial and error^51^. The reason is that the DNN model uses the backpropagation algorithm to modify the model's node weight since the ideal combination varies provisional on input and output variables. In the network structure scenario, the number of layers and the number of nodes is specified. In hyper-parameter tuning, optimal hyper-parameter components (i.e., optimizer, activation function, batch size, and epoch) are set through several combinations. The optimizer controls the stability and the speed of data learning of the model and the activation function determines how to define the least cost function. The batch size sets the data learning unit of the model to designate efficient learning and the epoch determines the number of data learning for the model. Dropout is a kind of regularization penalty to avoid the performance of the deep learning model from falling through overfitting^51,52^. This study deliberated the limited amount of data. The dropout was set to be 0 or 0.2 with three layers. Simulation was performed to find the optimal combination. Adaptive Moment Estimation (Adam) was adopted as an optimizer. Adam Method is a kind of optimization algorithm with the theory of moment of a stochastic objective function. This algorithm is an extensively accepted optimizer because of its convenience and versatility in computation^53^. The Rectified Linear Unit (ReLu) function is agreed as an activation function. ReLu is a function developed to complement the current sigmoid function. It is an activation function that alters the output according to the value greater than or equal to 0^54^. In addition, the epoch was set to be 1000 and the batch was set to be 5.

**Figure 2:**
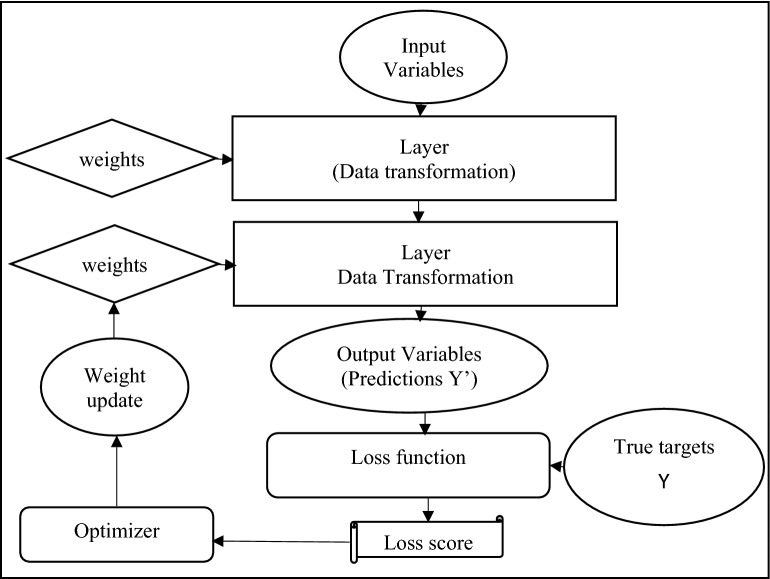
DNN backpropagation algorithm.

The above algorithm (Fig. 2) begins with the final loss and operate backward beginning from the higher layers to the lower layers, using chain rule to calculate the effect of each variable on the value of the loss^55^. Table 3, displays MAE and RMSE values of the learning results according to the network structure scenario and dropout. Among these values, the scenario having the minimum value was determined as the final network structure scenario. The scenario with minimum values of MAE and RMSE was selected as the final model. Learning results normally exposed a smaller loss function when dropout was 0 than when dropout was 0.2. Also, in a scenario where the number of hidden layer nodes was 400-400-400, both MAE and RMSE showed the smallest values. The larger the number of hidden layer nodes, the larger the MAE and RMSE. As a result, in the network structure scenario of the finishing model, the number of hidden layer nodes was 400-400-400 and the dropout was 0.

**Table 3:** Learning results.

Network structure scenario	Dropout (0)		Dropout (0.2)	
	MAE	RMSE	MAE	RMSE
5-5-5	0.906	1.124	1.127	1.406
10-10-10	0.697	0.897	0.891	1.146
25-25-25	0.558	0.672	0.720	0.855
50-50-50	0.376	0.580	0.557	0.756
75-75-75	0.351	0.581	0.539	0.76
100-100-100	0.350	0.505	0.534	0.675
200-200-200	0.377	0.516	0.562	0.709
300-300-300	0.336	0.473	0.531	0.656
400-400-400	0.331	0.448	0.529	0.625
500-500-500	0.353	0.449	0.541	0.629
600-600-600	0.338	0.458	0.534	0.641
700-700-700	0.353	0.465	0.536	0.641
800-800-800	0.483	0.475	0.668	0.681
900-900-900	0.346	0.514	0.537	0.704
1000-1000-1000	0.354	0.493	0.538	0.671

### Validation of model

The final network structure and hyper-parameters are displayed in Table 4. For verification of the final model, MAE and RMSE values of verification data and test data were estimated. That is to check whether the model will perform efficiently on a new data set^56^. Simulation results, are summarized in Table 5. As shown in Table 5, MAE and RMSE values of the validation data were 0.402 and 0.349, respectively. MAE and RMSE values of the test data were 0.535 and 0.456, respectively. Therefore, differences between values of validation and test data were not outsized and the problem of overfitting the model was found to be trivial. For additional model validation, a multiple regression analysis (MRA) model was developed using the multiple regression analysis method with the same data. Multi-regression method is commonly applied to continuous outcome variable with multiple covariates^57,58^. MAE and RMSE values were assessed to compare results of the DNN model and the MRA model. The MRA model was established using the IBM Statistical Package for the Social Sciences (SPSS) version 23. Multiple regression analysis is a regression analysis method that estimates the fundamental correlation between variables utilizing a statistical method. It is generally agreed in the field of prediction^59^. As a result, paralleling result values of the DNN model and the MRA model, the DNN model exhibited a lesser prediction error rate of 41.3% in MRA and 46.0% in RMSE than the MRA model. This better performance of backpropagation algorithm compared to MRA model was also noticed by Wei and Yang^56^.

**Table 4:** Final network structure and hyper-parameter.

Set	Configuration	Feature
Network structure	Node	3
	Layer	400-400-400
Hyper parameter	Activation function	Rectified linear unit function
	Optimizer	Adaptive moment estimation method
	Batch size	5
	Epoch	1000
	Dropout	0

**Table 5:** Model comparison results.

Model	Validation		Test	
	MAE	RMSE	MAE	RMSE
DNN	0.402	0.349	0.535	0.456
MRA			0.912	0.844
DNN/MRA (%)			− 41.3%	− 46.0%

## Discussion

This study proposed a framework for developing a model to predict financial losses of apartment construction sites employing the DNN algorithm, one of deep learning algorithms. For data collection, data on financial loss occurring at actual apartment construction sites were collected utilizing claim payout records of an insurance company and variables were gathered. With the purpose of developing an optimal model for learning input and output variables, network scenarios and hyper-parameters were firm over and done with trial-and-error methods. The final DNN model was verified by comparing prediction results of validation and test data and additionally by matching prediction results with the MRA model. As a result of model contrast, the DNN model presented 41.3% lesser MAE and 46.0% lower RMSE than the MRA model, indicating that the DNN model had a lower prediction error rate than the MRA model. In addition, it was demonstrated that DNN was a further consistent model for expecting the financial loss of an apartment construction project because it displayed a lower prediction error rate than the MRA model. This is because the nonparametric model DNN is more adequate than the parametric MRA model to analyze the uncertainty and nonlinearity of financial loss data of apartment construction sites^59,60^.

Through the developed model of this study, the client or contractor of an apartment construction project can predict the loss of a construction site or use the framework of this study to develop a deep learning prediction model according to situations of other apartment construction projects. In addition, since the prediction result showed a lesser prediction error rate than the existing model, more accurate and robust prediction will be possible. Based on this loss prediction, the client of an apartment construction project can make various investments to reduce and prevent expected loss. For example, before starting the construction of an apartment project, the client can prepare various measures to reduce and prevent expected losses and actively invest in facilities. Besides, it is possible to prepare for unexpected losses by preparing an emergency reserve in consideration of the expected loss at the phase of financial planning. The project owner can set a plan for financial risk in consideration of owner’s asset size and risk preference and utilize it to accomplish economic soundness and continuity of the project. This model can be helpfully adopted to expand insurance coverage and purchase special contracts to transfer the risk of additional economic loss. Furthermore, it can be potentially applied at an appropriate rate level for insurance premiums for current apartment construction projects or for future construction projects. By predicting, preventing, reducing, and transferring these economic losses, it will be conceivable to reduce economic losses that may occur in the future.

In addition, the developed model can be employed in a model to analyze and predict financial loss in other industrial fields or industrial sites in the future. Thus, it can be widely used in the private or public sector. Besides, abnormal climate and severe weather (e.g., heavy rain, heavy snow, hurricane, hail, lightning, tornado, etc.) caused by climate change due to global warming are global challenges that affect various fields such as society and economy^61–64^. Under these circumstances, there have been many studies to find the relationship between construction projects and natural disasters. For instance, in hurricane-stricken areas, it has been shown that the distance between the hurricane's strong wind and the shoreline is sturdily related to the damage to buildings caused by the hurricane^65,66^. The present study confirmed that natural disaster risk indicators such as elevation, flood risk, and strong wind risk were closely related to the risk of financial loss at construction sites, supporting findings of previous studies. Moreover, natural disasters are expected to increase in severity and frequency owning to climate change. Thus, they can be established as events that have a profound negative impact on construction projects^7,15^. Results and framework of this study can be utilized to prevent and reduce financial loss from natural disasters, ultimately achieving financial reliability of construction projects. Furthermore, considering the impact of the construction site due to natural disasters, it is necessary to excavate additional variables centered on the construction site, such as weather and geographic location of the construction site. Consequently, in the future, it is necessary to develop an additional risk assessment model that considers other damages (e.g., safety accident, damage to human life) at construction sites that reflect these additional indicators.

Furthermore, ICT (Information and Communications), sensors, and IoT (Internet of Things) related technologies and devices are being actively introduced into construction sites recently. Since these technologies and devices will produce a huge amount of big data, this study will be a basic research for studying such big data. However, this study collected data from claim payout records of apartment construction sites that occurred at a Korean insurance company. Therefore, additional research is needed to perform comparative analysis and verification through additional data collection from other insurers and various countries. In addition, to advance the model, extra research is necessary based on increased amount of data through the collection of additional data and qualitative increase through identification of added variables. Moreover, since this study collected and analyzed data from only apartment construction sites, further research on other types of construction projects is needed. More research is also needed to determine risk indicators through data collection of other types of construction projects and compare results and frameworks with this study.

Into the bargain, this study reduced prediction errors by building a model using the DNN algorithm. However, interrelationships and weights between nodes are in the black box of the DNN algorithm. This shows that AI can unilaterally provide results of predictions with a fatal limitation in logically explaining the basis and process for drawing the results, which may negatively affect the reliability and fairness of future models. Therefore, in future AI model research, users need to be provided with explanations about the decisions and results of AI models. Thus, technologies such as explainable AI (XAI) that can increase users’ understanding and confidence in AI models through a glass box rather than a black box introduction are needed. XAI is one of artificial intelligence technologies that can explain to users about decision-making and causality as well as classification and prediction through existing big data analysis algorithms^67^. By developing a model through XAI, it will be possible to understand the results and decision-making of the AI model and to trust the results, thereby relieving users' anxiety about black box results and improving the insight into big data and models.

In this study, limitations in its prediction and usage due to the data limitation has been enhanced. More specifically, it is a specialized predictive model for only safety accidents in apartment construction projects by collecting only safety accidents data that occurred in apartment construction, a major residential type in Korea. In addition, based on the collected safety accident data, more variables related to safety accidents were collected and used in the predictive model. Therefore, by using this model, by predicting possible safety accident losses in apartment construction, construction entities such as insurance companies, construction companies, and ordering companies can easily and accurately predict the amount of safety accident losses, which can be used for safety accident execution or budgeting. Also, in DNN model setting, when hyper-parameter tuning is performed, more optimal hyper-parameter components (i.e., optimizer, activation function, batch size, and epoch) are tested based on the past study to find the optimal combination, and more network structure scenarios were tested to improve the model's completeness. Hence, compared to previous similar researches, this study provides more advanced and significantly enhanced model focusing on safety accidents of specific occupancy.

## Conclusions

Recently, the scale of financial loss in construction works is rapidly increasing due to increasing sizes of construction projects, the complexation of buildings, and the increase in urban construction. Moreover, increases in the frequency and severity of natural disasters due to climate change are emerging as new risk factors of construction works, thus increasing the uncertainty of construction works. For well-organized and maintainable management of construction projects, a delicate and robust loss prediction model is essential to predict financial losses and establish strategies for risk management such as prevention, reduction, and transfer of risks. Therefore, this study recommended a framework for developing a predictive model using the deep learning algorithm based on loss data generated at apartment construction sites.

In this study, a model using the DNN algorithm was developed for predicting the cost of financial losses at apartment construction sites. It was verified through comparison with other models. As a result of verification, the DNN model developed in this study showed a lower prediction error than the existing model in predicting financial losses at apartment construction sites. It will contribute to the prediction of financial losses at construction sites. Therefore, it can be an effective method to predict financial losses of apartment construction sites using results and framework of this study. It is also a reference material for reducing financial management and loss risk of future construction projects. In addition, by using results and framework of this study, it is judged that it can be widely applied to other industrial sites, other research fields, and other construction types of projects. Thus, it will ultimately lead to decreased financial losses of industrial sites. Besides, it will be possible to upgrade to a reliable model through continuous research such as continuous data collection, addition of variables, and supplementary verification of the model. Additionally, risk factors displayed in this study (e.g., construction characteristics, environment, natural disasters, etc.) could be used as reference materials for predicting financial losses of government agencies or the private sector. For example, government agencies can use results of this study to prepare systems and standards for preventing and reducing damage at apartment construction sites. For areas vulnerable to natural disasters, insurance companies can use results and framework of this study to manage risks by setting various insurance conditions such as portfolio composition, probable maximum loss, event limit, and appropriate premium according to the company’s risk appetite and asset size.
